# Individual, occupational, and dysphonia-related factors in operational and administrative military police officers

**DOI:** 10.1590/2317-1782/e20240230en

**Published:** 2026-03-27

**Authors:** Stephanie Mayra de Moraes, Letícia Caldas Teixeira, Adriane Mesquita de Medeiros

**Affiliations:** 1 Programa de Pós-graduação em Ciências Fonoaudiológicas, Departamento de Fonoaudiologia, Universidade Federal de Minas Gerais – UFMG - Belo Horizonte (MG), Brasil.

**Keywords:** Police Professionals, Vocal Health, Voice Disorders, Epidemiological Surveys, Working Conditions

## Abstract

**Purpose:**

To analyze occupational, individual, and dysphonia-related factors in military police officers in operational and administrative service.

**Methods:**

Observational, cross-sectional study with 482 police officers (77 administrative and 405 operational) from Belo Horizonte, Brazil. Data were collected through a questionnaire, sent via the Internet, with sociodemographic, occupational, and dysphonia-related questions, as well as the Brazilian Dysphonia Screening Tool (Br-DST) and the Job Stress Scale (JSS). The sample included police officers on active duty, from the enlisted personnel, who were not in military training courses. Those who did not respond to the survey by the end were excluded. Descriptive and association analyses between categorical variables were performed using the chi-square or Fisher's exact test.

**Results:**

Police officers in administrative and operational services had different police service time, perception of social support at work, threat or aggression at work, and raised voice in intense noise. The probability of moderate/high dysphonia was similar for police officers on operational and administrative duty. Approximately 18% of officers reported straining to speak, and 9% reported hoarseness. Absence from work due to voice problems was low in both service levels.

**Conclusion:**

Police officers on operational duty reported shorter service time, greater exposure to threats and physical aggression, raised voice due to intense noise, and low social support at work. Those working in administrative roles had opposite characteristics, suggesting a less health-unfavorable work environment.

## INTRODUCTION

The Brazilian Military Police (MP), disciplined in operational portfolios, is essential for security and public order^(1)^. The MP force is divided into two main classes (enlisted personnel and commissioned officers) and performs duties in operational and administrative service. Those in operational service respond to incidents received through emergency calls, conduct visible and repressive patrols against violent crime, register police reports, and perform other activities, while those in administrative service are responsible for the strategic management of all logistical and human resources for MP tasks^(2)^, including the control of emergency calls (made to phone number 190).

Thus, operational and administrative police officers are exposed to different work demands, which imply specific occupational and vocal risks, these still little explored in the scientific literature. In general, the daily routine of military service is marked by task overload, short deadlines^(3)^, low decision-making autonomy, and limited social support at work^(4)^. These factors pose barriers to quality of life and a healthy occupational environment, which are associated with stress levels frequently higher than those found in other professional categories^(5,6)^.

Being an MP officer knowingly demands physical and mental effort. Functional aging in the MP is premature and appears to be related to working conditions^(7)^. Over the years, occupational stressors of operational activity, such as continuous surveillance and frequent exposure to conflict, aggression, and violence in the community, tend towards morbidities related to police activity^(3)^. These aspects contribute to absenteeism and favor the migration of police officers to administrative service^(7)^, considered less strenuous and with reduced levels of exposure to physical and psychosocial risks than operational service.

The military voice is an essential tool for them to perform their duties, being important to communicate with the public, command, control adverse situations, and impose authority, especially in situations of conflict and tension. However, scientific research has seldom addressed the working conditions of military personnel in administrative and operational service and their possible consequences for vocal health.

Dysphonia, characterized by impairments that hinder natural voice production, may be related (though not exclusively) to environmental and organizational factors in the work setting^(8)^. These include the need to speak louder than the occupational noise, prolonged voice use, and vocal adjustments required in different environments^(4)^. These factors may manifest differently for police officers in operational and administrative services. A study conducted in an emergency call center of a public agency, with the participation of firefighters, MP officers, and civilians who worked as teleoperators in the administrative service, showed an association between the self-reported perception of noise and stressful work with the vocal condition of the teleoperators^(9)^.

Although police officers may need to use gradual levels of physical force in operational service, according to the aggressor's behavior, verbalization is the first form of intervention^(10,11)^. MP officers are not included among occupational voice users^(4)^; nonetheless, communicating and negotiating are part of their routine service, and responding to vocal demands in the police officer's work can be detrimental to their voice.

Hence, this study aimed to analyze the occupational, individual, and dysphonia-related factors in MP officers in operational and administrative service.

## METHODS

This is an observational, cross-sectional, analytical study, based on primary data obtained through a web survey.

The research was approved by the Human Research Ethics Committee under approval number 5.179.501 and by the MP’s Research and Postgraduate Center, protocol number 202204051646483-2204.

The MP is organized into two occupational frameworks – commissioned officers and enlisted personnel. The population of this study consisted of MP enlisted personnel, referred to with the umbrella term of police officers. Enlisted personnel encompass those occupying increasing hierarchical ranks, starting with soldiers, followed by corporal, sergeant, and sub-lieutenant, which is the highest rank within this category. A police officer may serve in operational or administrative roles regardless of their hierarchical rank.

The total population of MP officers in Belo Horizonte, Brazil, was 4,107 during the data collection period. The study used the following formula for the sample size calculation: n = N x Z^2^x p(1-p)/ Z^2^ x p (1 –p) + e^2^ x N – 1, in which n is the sample size obtained through calculation; N is the total research population; Z is the indicated deviation from the mean value acceptable to reach the confidence level; and p is the estimated proportion of dysphonia in the investigated population. The sample was estimated to be 351 participants, considering a 3% sampling error, a 95% confidence level, a 50% estimated outcome prevalence, and an additional 20% loss.

The inclusion criteria were MP officers on active duty at the institution under study, belonging to the enlisted ranks, and not being in a military training course. Participants who did not respond to the survey by the end were excluded.

A pilot study was conducted prior to data collection, using an online questionnaire administered to 10 military personnel to assess the suitability of the questions in terms of form and content and to estimate the average time required to complete it, which ranged from 5 to 10 minutes.

Data were collected through a questionnaire sent three times via the institutional intranet messaging channel between May and June 2022 to 4,107 MP officers. Of these, 482 (12%) agreed to participate in the survey. The questionnaire was sent to the MP officers via the institutional intranet, containing several questions divided into three sections, as described below:

1. The Brazilian Dysphonia Screening Tool (Br-DST)^(12)^, a self-assessment protocol recently validated in its Portuguese version^(13)^. The Br-DST analysis considers the answers to the questions: 1. “I feel I have to strain to make my voice come out,” and 2. “My voice is hoarse.” A positive answer to both questions suggests a high probability of dysphonia, indicating the need for a complete clinical evaluation. A positive answer only to question two (hoarse voice) suggests a moderate probability of dysphonia and the need for vocal guidance and monitoring through evaluation. Finally, a positive answer only to question one (straining to make the voice come out) or a negative answer to both questions indicates a low probability of dysphonia, suggesting the need for voice care guidance^(12,13)^.
2. Sociodemographic and absenteeism questions, including sex, age, marital status, education level, and absence from work due to voice problems in the previous 12 months. Age was collected as full years and dichotomized according to the mean/median (38 years).
3. Occupational aspects, including actual MP working time; voice raised due to intense occupational noise; and three questions from the list of occupational traumatic events for emergency professionals, validated in the Portuguese-translated version^(14)^, addressing the frequency with which the professional was exposed to 1. threat of physical aggression at work; 2. personal physical aggression at work; and 3. threat or physical aggression against a coworker, in the previous 12 months. The 17-question Job Stress Scale (JSS)^(5)^, validated for Portuguese, was used to analyze the following dimensions of work organization: demands (exposure to pressure, such as deadlines and speed of tasks [four questions] and work process [e.g., antagonistic demands, one question]) and control (professional skills, competence, and decision-making [six questions]). The response options for these questions are organized on a 4-point Likert scale: often, sometimes, seldom, and never/almost never. The responses for each work organization dimension were summed to form the Demand-Control Model, and the result was dichotomized using the median as a cutoff. After dichotomizing each dimension, the four quadrants of the Demand-Control Model were formed: low burnout (low demand and high control); passive work (low demand and low control); active work (high demand and high control); and high burnout (high demand and low control)^(5,15,16)^. The other JSS dimension refers to social support at work, related to assistance and approval from superiors and colleagues (six questions). After summing the responses and considering the median of the results, social support at work was dichotomized into low and high support^(5,15,16)^.

All categorical variables were described for data analysis using absolute and relative frequencies for the total number of participants, and separately for each type of service performed (administrative and operational). The explanatory variable was police officers in administrative or operational service. The other variables investigated were considered response variables. Bivariate analyses between the response and explanatory variables were performed using the chi-square test or Fisher's exact test. The latter was an alternative to the former in cases where the expected frequencies in the contingency table cells were less than five. The significance level was set at 5%. Data analysis was performed using Stata® statistical software, version 16.1.

## RESULTS

Among the MP officers in this study (n = 482), 77 (16%) belonged to the administrative service, and 405 (84%) belonged to the operational service. The predominance among the total number of police officers was of males (84.6%); age range from 23 to 38 years (52.3%); marital status with a partner (70.6%); and undergraduate/bachelor's degree as the highest level of education (52.7%) (Table 1). Most police officers had 11 to 20 years of service (55.8%) and reported that they sometimes needed to raise their voice due to intense noise at work (59.0%). Episodes of physical aggression more than once a month were reported by 7.4%, and threats of physical aggression were reported by 20.5%, both with the police officer as the victim. Threats or aggressions against colleagues were reported by 19.5% of the police officers (Table 2).

**Table 1:** Descriptive and association analysis of sociodemographic and voice-related issues according to the service performed by military police officers (Belo Horizonte, 2022)

**_Variables_**	**_Total n (%)_**	**_Administrative service_**		**_Operational service_**		**_p-value_**
		**_n_**	**_%_**	**_n_**	**_%_**	
_Sex_						
_Females_	_74 (15.4)_	_17_	_22.1_	_57_	_14.1_	_0.074_ _1_ ^_*_^
_Males_	_408 (84.6)_	_60_	_77.9_	_348_	_85.9_	
_Age range (in years)_						
_23 to 38_	_252 (52.3)_	_31_	_40.3_	_221_	_54.6_	_0.021_ _1_ ^_**_^
_39 to 54_	_230 (47.7)_	_46_	_59.7_	_184_	_45.4_	
_Marital status_						
_Married or other form of union_	_334 (70.6)_	_50_	_66.7_	_284_	_71.4_	_0.413_ _1_
_No partner_	_139 (29.4)_	_25_	_33.3_	_114_	_28.6_	
_Education level_						
_Elementary to high school_	_131 (27.2)_	_16_	_20.8_	_115_	_28.4_	_0.387_ _1_
_Bachelor’s degree_	_254 (52.7)_	_44_	_57.1_	_210_	_51.8_	
_Postgraduate degree_	_97 (20.1)_	_17_	_22.1_	_80_	_19.8_	
_“I feel I have to strain to make my voice come out” (Br-DST)_						
_No_	_396 (82.2)_	_64_	_83.1_	_332_	_82.0_	_0.810_ _1_
_Yes_	_86 (17.8)_	_13_	_16.9_	_73_	_18.0_	
_“My voice is hoarse” (Br-DST)_						
_No_	_437 (90.7)_	_70_	_90.9_	_367_	_90.6_	_0.936_ _1_
_Yes_	_45 (9.3)_	_7_	_9.1_	_38_	_9.4_	
_Probability of dysphonia (Br-DST)_						
_Low_	_437 (90.7)_	_70_	_90.9_	_367_	_90.6_	_0.241_ _1_
_Moderate_	_26 (5.4)_	_2_	_2.6_	_24_	_5.9_	
_High_	_19 (3.9)_	_5_	_6.5_	_14_	_3.5_	
_Absence from work due to voice problems_						
_No_	_469 (97.3)_	_58_	_100.0_	_254_	_95.1_	_0.101_ _2__*_
_Yes_	_13 (2.7)_	_0.0_	_0.0_	_13_	_4.9_	

1 Chi-square test;

2 Fisher's exact test;

* p-value < 0.20;

** p-value < 0.05

Caption: Br-DST = Brazilian Dysphonia Screening Tool; n = absolute frequency; % = relative frequency

**Table 2:** Descriptive and association analysis of work-related issues according to the service performed by military police officers (Belo Horizonte, 2022)

**_Variables_**	**_Total n (%)_**	**_Administrative service_**		**_Operational service_**		**_p-value_**
		**_n_**	**_%_**	**_n_**	**_%_**	
_Service time in the military police (in years)_						
_1 to 10_	_134 (27.8)_	_07_	_9.1_	_127_	_31.4_	_<0.001_^_1_ _**_^
_11 to 20_	_269 (55.8)_	_52_	_67.5_	_217_	_53.6_	
_21 to 30_	_79 (16.4)_	_18_	_23.4_	_61_	_15.0_	
_Raising the voice due to loud noise at work_						
_Never/rarely_	_65 (13.4)_	_17_	_22.1_	_48_	_11.8_	_<0.001_ _1__**_
_Sometimes_	_284 (59.0)_	_52_	_67.5_	_232_	_57.3_	
_Often_	_133 (27.6)_	_08_	_10.4_	_125_	_30.9_	
_Personal physical aggression at work_						
_Never/less than once a month_	_446 (92.6)_	_74_	_96.1_	_372_	_91.9_	_0.141_ _2_ ^_*_^
_More than once a month_	_36 (7.4)_	_03_	_3.9_	_33_	_8.1_	
_Threat of physical aggression at work_						
_Never/less than once a month_	_383 (80.5)_	_73_	_94.8_	_310_	_76.5_	_<0.001_ _2__**_
_More than once a month_	_99 (20.5)_	_04_	_5.2_	_95_	_23.5_	
_Threat or physical aggression towards a coworker_						
_Never/less than once a month_	_388 (80.5)_	_69_	_89.6_	_319_	_78.8_	_0.028_ _1__**_
_More than once a month_	_94 (19.5)_	_08_	_10.4_	_86_	_21.2_	
_Social support at work_						
_Low_	_249 (51.7)_	_24_	_31.2_	_225_	_55.6_	_<0.001_ _1__**_
_High_	_233 (48.3)_	_53_	_68.8_	_180_	_44.4_	
_Demand-Control Model_						
_Low burnout_	_100 (20.8)_	_20_	_26.0_	_80_	_19.8_	_0.499_ _1_
_Passive work_	_99 (20.5)_	_17_	_22.0_	_82_	_20.2_	
_Active work_	_150 (31.1)_	_23_	_30.0_	_127_	_31.4_	
_High burnout_	_133 (27.6)_	_17_	_22.0_	_116_	_28.6_	

1 Chi-square test;

2 Fisher's exact test;

* p-value < 0.20;

** p-value < 0.05

Caption: n = absolute frequency; % = relative frequency

Being a police officer in operational or administrative service was not statistically significantly associated with having to strain to speak, having a hoarse voice, absenteeism due to voice problems, or the likelihood of dysphonia. Three out of every 100 police officers reported having been absent from work due to dysphonia in the previous 12 months, these being from operational service (Table 1).

The following variables were statistically significantly associated with being a police officer in operational or administrative service: time in the MP; raising one's voice due to intense noise at work; personal physical aggression; threat of physical aggression; threat or physical aggression against a colleague; and social support at work (Tables 1 and 2).

## DISCUSSION

The results suggest that operational and administrative police officers are exposed to different working conditions. However, the frequency of reports of hoarseness and effort to produce a clear voice was similar between the two groups: 18% of operational police officers and 17% of administrative officers reported effort to produce a clear voice, and 9% reported a hoarse voice. Vocal effort was reported more frequently than hoarseness, which may indicate intense vocal demands even without a clearly perceived change in vocal quality.

Vocal effort is a multidimensional concept that can indicate increased loudness, muscle tension, and contextual factors such as background noise, distance from the speaker, cognitive load, and emotional aspects^(17)^. Auditory symptoms such as hoarseness, deep voice, and weak voice are mainly related to excessive and prolonged voice use, with overload on the vocal tract^(18)^.

A higher proportion of administrative police officers had a high probability of dysphonia (6.5%) than operational officers (3.5%), although without statistical significance. The moderate probability of dysphonia was higher among operational officers (5.9%). The data did not confirm the initial hypothesis of a higher risk of dysphonia among operational officers, but the results raise relevant questions about the distinct characteristics of voice use between the two services. It is speculated that factors such as the continuous voice use in administrative service in tasks like call center work may contribute to the risk of voice disorders. On the other hand, operational service involves more intense voice use in noisy environments and under physical and emotional stress, which can also impact vocal health.

In general, according to the Br-DST validation, 9% of police officers in this study had a moderate/high probability of dysphonia and needed to undergo a complete vocal assessment to confirm the dysphonia diagnosis. A study with 442 firefighters found a moderate/high probability of dysphonia of 6.12%^(19)^. Both studies used Br-DST, which is sensitive in capturing the probability of dysphonia, presenting a higher accuracy rate in classifying this condition than other self-assessed dysphonia screening instruments^(13)^. The prevalence of dysphonia found in this study was similar to that of other studies with North American adults^(20,21)^. However, the instruments used for dysphonia screening were different, and the results should be compared with caution.

The predominant factors among police officers in operational service were shorter service time, greater exposure to threats or physical aggression, raising their voices in intense noise, and less social support at work.

The results showed a higher proportion of administrative police officers with more than 10 years of MP service and operational police officers with less time in their careers. The greater health strain on the military personnel, as well as the fearsome evidence of personal aggression in operational work^(3)^ may justify this finding. The study verified the tendency for police officers to be reassigned to administrative service due to physical and psychological strain in operational service^(7)^. Our results reflect a more stable and less physically demanding work environment for police officers in administrative service, unlike operational service, which is associated with greater exposure to adverse conditions. These administrative characteristics may be more attractive to women and to police officers with more years of service due to gender issues and greater physical and mental strain over the years of work^(7)^.

This study indicated that police officers on operational duty had a greater need to frequently raise their voices due to intense occupational noise. Workplace noise can negatively influence vocal health, especially when combined with other individual and organizational risk factors^(22)^. Given exposure to high street noise, military personnel are at higher risk of developing hearing loss and tinnitus^(23)^. Moreover, age, length of service, and exposure to gunfire are also predictive of hearing health problems^(24)^.

From this perspective, speaking loudly in intense noise proved to be more frequent in operational service, given its interference with MP officers’ verbal orders and general communication. This study did not aim to analyze the relationship between voice and noise, but it identified a 30.9% prevalence of frequent need to raise the voice in occupational noise reported by police officers in operational service, in contrast to 10.4% of police officers in administrative service.

Epidemiological surveys investigating the health-work relationship have prioritized occupational stress factors, analyzed according to the demand-control model^(25)^. The demand-control model in this study was not statistically significantly associated with the type of service performed by police officers. However, the proportion of those with high burnout (29%) was higher in operational than in administrative service (22%).

A study with operational police officers showed that this service has more intense and exhausting demands, especially among professionals of lower hierarchical rank^(26)^. Thus, social support at work, whether from colleagues or from bosses and supervisors, influences health and job satisfaction, being essential for maintaining and promoting the health of workers^(27)^. In line with this evidence, our results indicated low levels of social support, mainly among police officers on operational duty, which may worsen their psychosocial conditions.

A study found that police officers on operational duty have a higher prevalence of occupational stress, are absent from work more often, and have fewer social ties, among other behaviors that contribute to professional illness^(28)^. The relationship between firefighters’ work demands and psychiatric morbidity was attenuated with high levels of social support and job control^(28)^. Teachers with low social support at work were more likely to have voice-related work limitations than those with high social support at work^(29)^.

This study found a low frequency of absenteeism due to voice problems, restricted only to police officers on operational duty. The prevalence of absences was lower than that of adults in the general population (9.2%) during the 12-month reference period^(20)^. Another study showed that 80% of call center operators at an emergency call center who were absent from work due to voice problems at some point in their careers had voice complaints^(9)^. MP officers may continue working even with dysphonia, which may result in a late search for professional help (speech-language-hearing pathologist or otolaryngologist), worsening the problem over time. Aggravated dysphonia can hinder the work of police officers on operational duty, especially for activities with higher vocal demands.

Exposure to workplace violence was more prevalent among police officers on operational duty, as expected. The threat of physical aggression at work was the most prominent (23.5%), indicating a greater stress factor for this group and potentially leading to increased voice intensity and body tension. A study with basic education teachers showed a relationship between limitations at work due to self-reported voice disorders and exposure to verbal violence perpetrated by students^(29)^. However, another study with teachers did not statistically significantly associate self-reported voice disorders with violent situations^(30)^.

It is important to note that this study had a non-probabilistic sample, meaning that the results cannot be generalized to the target population. The results indicate a need for greater understanding of the influence of occupational noise, physical threats or aggression, and social support as possible risk factors for dysphonia among MP officers. Future studies should address these factors to elucidate how they relate to these professionals’ vocal health. Furthermore, the smaller proportion of police officers in administrative service in this study's sample is not a sampling error; rather, it reflects the composition of the MP of Minas Gerais, Brazil. The unequal sample sizes between the administrative and operational groups may imply interpretative limitations. However, it is believed that the analysis offers relevant contributions to understanding the differences in working conditions and the probability of dysphonia among police officers in the two types of service.

The healthy worker effect is a common bias in occupational studies^(31)^ and may be relevant in interpreting the results of this research. It can occur because less healthy police officers or those with conditions that prevent them from performing more physically demanding activities tend to be transferred to administrative duties or leave work altogether, while healthier ones remain or are allocated to operational duties. This may have influenced the voice-related results in this study, considering that vocal symptoms can reflect various health conditions, such as respiratory, gastric, and mental problems, among others^(32)^.

The results of this study lack external validity and should be interpreted with caution. MP officers are not traditionally recognized as occupational voice users, which may lead them to underestimate or not identify vocal symptoms. Moreover, police officers tend to migrate from operational to administrative roles throughout their careers. These factors may impact the detection of differences in the probability of dysphonia between police officers in operational and administrative roles.

This study was limited by the fact that the questionnaire was sent to participants by the military institution, which makes it difficult to control the application method. Studies using web surveys offer advantages such as remote data collection, lower cost, and greater ease of implementation^(33)^. The exact number of users reached was ascertained, whereas the number of those who viewed the email remained unknown. The response rate was low, and it was not possible to evaluate non-response – i.e., the information necessary to determine whether non-participants were similar to participants^(33)^. On the other hand, the use of previously validated questionnaires was an excellent decision in the pursuit of consistency and accurate measurements. Testing the online questionnaire version before starting the study to assess comprehensibility and the ease and time of response was another positive point^(33)^.

The results indicated occupational stressors that may or may not be associated with dysphonia in MP officers. Future studies should analyze the factors associated with dysphonia in administrative and operational MP officers, considering the distinct working conditions between the groups. Actions to prevent dysphonia and promote vocal health among MP officers are necessary, focusing on raising awareness about risk factors for dysphonia and advising on how to avoid or minimize them. Such actions should be accompanied by institutional measures that improve working conditions, such as controlling environmental noise, valuing social support within teams, adjusting workload, and offering training on healthy voice use in the context of police activities.

## CONCLUSION

MP officers on operational duty have shorter service times, higher reports of exposure to physical threats or aggression, increased voice volume due to intense noise in the workplace, and low social support at work, indicating unfavorable working conditions for their health. These factors can trigger or worsen voice disorders and other health problems.

The frequency of reported hoarseness and effort to produce the voice and the infrequent absences from work due to vocal problems were similar between police officers from both services. The probability of moderate/high dysphonia was similar among police officers in operational and administrative service, indicating that 9% of police officers should undergo a complete vocal assessment to confirm the diagnosis.
