# Supplementary material for: Individual, occupational, and dysphonia-related factors in operational and administrative military police officers
Source: Codas. 2026 Mar 27;38(2):e20240230. doi: 10.1590/2317-1782/e20240230en (PMC13075847; doi:10.1590/2317-1782/e20240230en)
Supplement: Translation

# Translation: Fatores individuais, ocupacionais e relacionados à disfonia em policiais militares operacionais e administrativos

**DOI:** 10.1590/2317-1782/e20240230pt

**Authors:** Stephanie Mayra de Moraes, Letícia Caldas Teixeira, Adriane Mesquita de Medeiros

**Affiliations:** 1 Programa de Pós-graduação em Ciências Fonoaudiológicas, Departamento de Fonoaudiologia, Universidade Federal de Minas Gerais – UFMG - Belo Horizonte (MG), Brasil.

**Keywords:** Profissionais da Polícia, Saúde Vocal, Distúrbios da Voz, Estudos Epidemiológicos, Condições de Trabalho

## Abstract

**Objetivo:**

Analisar os fatores ocupacionais, individuais e relacionados à disfonia em policiais militares do serviço operacional e administrativo.

**Método:**

Estudo observacional, transversal com 482 policiais (77 administrativos e 405 operacionais) de Belo Horizonte. Coleta de dados por meio de questionário, enviado via web com questões sociodemográficas, relacionadas à disfonia e ao trabalho. O Instrumento de Rastreio da Disfonia (IRDBR) e o protocolo Job Stress Scale (JSS) também foram utilizados. Foram incluídos policiais no serviço ativo, do quadro de servidores militares considerados como Praças, que não estavam em curso de formação militar. Foram excluídos quem não respondeu à pesquisa até o final. Realizaram-se análises descritiva e de associação entre variáveis categóricas por meio do teste Qui-Quadrado ou Exato de Fisher.

**Resultados:**

Os policiais em serviços administrativos e operacionais apresentaram diferenças quanto ao tempo efetivo na polícia, percepção de apoio social no trabalho, ameaça ou agressão no trabalho e elevação da voz na presença de ruído intenso. A probabilidade de disfonia moderada/elevada foi similar para o policial em serviço operacional e administrativo. Aproximadamente 18% dos policiais relataram fazer força para voz sair e 9%, voz rouca. Foram reduzidas as faltas ao trabalho por problema de voz em ambos os serviços.

**Conclusão:**

Os policiais no serviço operacional referiram menor tempo de serviço, maior exposição à ameaça, agressão física, elevação da voz devido ao ruído intenso e baixo apoio social no trabalho. Já os que atuam em funções administrativas apresentaram características opostas, sugerindo um cenário laboral menos desfavorável à saúde.

## INTRODUÇÃO

A polícia militar (PM), disciplinada em portfólios de atuação, é imprescindível para a segurança e a ordem pública^(1)^. Os policiais militares são agrupados em duas grandes classes: Praças e Oficiais e desempenham funções no serviço operacional ou administrativo. O policial no serviço operacional realiza o atendimento das ocorrências recebidas pelas chamadas de emergência, as patrulhas ostensivas e repressivas da criminalidade violenta, registra Boletins de Ocorrência Policial e outras atividades. O policial em serviço administrativo é responsável pela gestão estratégica de todo o recurso logístico e humano para a execução das tarefas da PM^(2)^, incluindo o controle das chamadas de emergências (ligações para o 190).

Assim, policiais operacionais e administrativos estão expostos a diferentes demandas de trabalho, o que implica em riscos ocupacionais e vocais específicos, esses ainda pouco explorados na literatura científica. De modo geral, o cotidiano do serviço militar é marcado por sobrecarga de tarefas, prazos curtos^(3)^, baixa autonomia decisória e limitado apoio social no trabalho^(4)^. Esses fatores atuam como barreiras à promoção da qualidade de vida e de um ambiente ocupacional saudável, que estão associados à níveis de estresse frequentemente superiores aos verificados em outras categorias profissionais^(5,6)^.

Sabe-se que a profissão de policial militar exige esforço físico e mental. O envelhecimento funcional na ocupação de policial militar é precoce e parece ter relação com as condições de trabalho^(7)^. Com o passar dos anos, os estressores ocupacionais da atividade operacional, como a vigilância contínua e a exposição frequente a situações de conflito, agressão ou violência na comunidade, demonstram uma tendência a morbidades relacionadas à atividade policial^(3)^. Esses aspectos contribuem para o absenteísmo e favorecem a migração dos policiais para o serviço administrativo^(7)^, considerado menos extenuante e com níveis reduzidos de exposição a riscos físicos e psicossociais em comparação ao serviço operacional.

A voz do militar é uma ferramenta essencial no exercício de suas funções. sendo importante na comunicação do policial com a população, na condução de comandos e no controle de situações adversas e na necessidade de imposição de autoridade, especialmente em situações de conflitos e tensão. No entanto, as condições de trabalho dos militares em serviço administrativo e operacional e suas possíveis consequências para a saúde vocal são pouco exploradas pelas pesquisas científicas.

A disfonia, caracterizada por alterações que impedem a produção natural da voz, pode estar relacionada — embora não exclusivamente — a fatores ambientais e organizacionais presentes no contexto laboral^(8)^. Entre eles estão a necessidade de sobrepor a fala ao ruído ocupacional, o uso prolongado da voz e os ajustes vocais exigidos em diferentes ambientes^(4)^. Esses fatores podem se manifestar de forma distinta para os policiais dos serviços operacional e administrativo. Um estudo realizado em uma central de atendimento a emergências de um órgão público, com a participação de bombeiros, policiais militares e civis que atuavam como teleoperadores no serviço administrativo, mostrou uma associação entre a percepção autorreferida de ruído e trabalho estressante com a condição vocal dos teleoperadores^(9)^.

No serviço operacional, embora a presença do policial tenha papel relevante entre os níveis graduais de uso da força física, conforme o comportamento do agressor, a verbalização é reconhecida como a primeira forma de intervenção^(10,11)^. Apesar de o policial militar não ser indicado como profissional da voz^(4)^, comunicar e negociar integram a rotina do serviço e a resposta à demanda vocal no trabalho do policial pode trazer prejuízos para a sua voz.

Desta forma, o objetivo deste estudo foi analisar os fatores ocupacionais, individuais e relacionados à disfonia em policiais militares no serviço operacional e administrativo.

## MÉTODOS

Trata-se de um estudo observacional, transversal e analítico, a partir de dados primários obtidos por meio de *Websurvey.*

A pesquisa foi aprovada pelo Comitê de Ética em Pesquisa com Seres Humanos sob o parecer: 5.179.501 e pelo Centro de Pesquisa e Pós-Graduação (CPP) da Polícia Militar, protocolo: 202204051646483-2204.

A PM é organizada por dois quadros ocupacionais, o de Patentes, relativos aos Oficiais, e o de graduações hierárquicas de Praças. A população deste estudo foi composta pelos Policiais Militares Praças. O Quadro de Praças é composto por militares que ocupam graduações hierárquicas crescentes, iniciando por soldado, seguido por cabo, sargento e subtenente, que representa o mais alto posto dentro dessa categoria. O policial pode atuar no serviço operacional ou administrativo independente da graduação hierárquica ocupada.

A população total dos policiais militares de Belo Horizonte foi de 4107 no período da coleta de dados e foi utilizada a seguinte fórmula para o cálculo amostral: n = N x Z^2^x p(1-p)/ Z^2^ x p (1 –p) + e^2^ x N – 1, em que: n é o tamanho da amostra obtida por meio do cálculo; N é o total da população pertencente à pesquisa; Z é o desvio indicado ao valor médio aceitável para que o nível de confiança seja atingido; e p é a proporção estimada da disfonia na população investigada. Identificou-se a amostra estimada de 351 participantes. Considerou-se o erro amostral de 3%, o nível de confiança de 95%, e o percentual de 50% para a ocorrência do desfecho (prevalência estimada) e acréscimo de perdas de 20%.

Os critérios de inclusão foram: ser policial militar, estar no serviço ativo da instituição do estudo, ser do Quadro de Praças e não estar em curso de formação militar. Foram excluídos participantes que não responderam à pesquisa até o final.

Antes da coleta de dados, foi realizado um estudo piloto com a aplicação do questionário *online* para dez militares com o objetivo de avaliar a adequação das questões em termos de forma e conteúdo, bem como estimar o tempo médio necessário para seu preenchimento, que variou entre cinco a dez minutos.

A coleta de dados foi realizada por meio de um questionário encaminhado por três vezes, pelo canal de mensagens da intranet institucional, no período de maio a junho de 2022, para os 4.107 policiais militares. Destes, 482 (12%) aceitaram participar da pesquisa. O questionário foi encaminhado aos policiais militares por meio da intranet institucional, contendo várias questões divididas em três blocos, conforme descrito a seguir:

1^o^) Instrumento de Rastreio da Disfonia (IRD^BR^)^(12)^, que é um protocolo de autoavaliação, com recente validação na versão em português^(13)^. A análise do IRD^BR^ considera a resposta das questões: 1. “Sinto que tenho que fazer força para a minha voz sair” e 2. “Minha voz é rouca.” A resposta positiva para ambas as perguntas sugere uma elevada probabilidade de disfonia, indicando a necessidade de uma avaliação clínica completa. A resposta positiva apenas para a pergunta dois (voz rouca) sugere uma probabilidade moderada de disfonia e a necessidade de orientação vocal e monitoramento da voz por meio de avaliação. E por último, a resposta positiva apenas para a pergunta um (fazer força para a voz sair), ou negativa para ambas as perguntas, indica uma probabilidade reduzida de disfonia, sugerindo a necessidade de orientação sobre os cuidados com a voz^(12,13)^.
2^o^) Questões sociodemográficas e de absenteísmo: sexo; idade; estado civil; escolaridade e ausência ao trabalho por problemas de voz nos últimos 12 meses. A variável idade foi coletada como idade completa em anos e dicotomizada segundo a média/mediana dos dados (38 anos).
3º) Aspectos relacionados ao trabalho incluindo: tempo de efetivo trabalho na PM; elevação da voz devido ao ruído intenso no trabalho e três perguntas da lista de eventos traumáticos ocupacionais para profissionais de emergência, validado na versão traduzida para o português^(14)^, que refere-se à frequência em que o profissional foi exposto à: 1. ameaça de agressão física no trabalho; 2. agressão física pessoal no trabalho e 3. Ameaça ou agressão física ao colega de trabalho, nos últimos 12 meses. O protocolo *Job Stress Scale* (JSS)^(5)^, validado para o português, com 17 questões foi utilizado para analisar as seguintes dimensões da organização do trabalho: o aspecto da demanda, relacionado à exposição de pressão, como prazos e velocidade de tarefas (quatro questões) e ao processo de trabalho, como demandas antagônicas (uma questão); o controle, sobre habilidade profissional, competência e decisão (seis questões). Todas as questões apresentam opções de respostas organizadas em escala *Likert* de quatro pontos: ‘frequentemente’, ‘às vezes’, ‘raramente’ e ‘nunca ou quase nunca’. Para a formação do Modelo Demanda-Controle foi feito o somatório das respostas para cada dimensão da organização do trabalho e o resultado dicotomizado a partir da mediana como ponto de corte. Após dicotomizar cada dimensão, foram formados os quatro quadrantes do Modelo Demanda-Controle: baixo desgaste (baixa demanda e alto controle); trabalho passivo (baixa demanda e baixo controle); trabalho ativo (alta demanda e alto controle) e alto desgaste (alta demanda e baixo controle)^(5,15,16)^. A outra dimensão do JSS refere-se ao apoio social no trabalho, relacionada à assistência e aprovação da chefia e dos colegas (seis questões). Após a soma das respostas e considerando a mediana dos resultados, o apoio social no trabalho foi dicotomizado em baixo e alto apoio^(5,15,16)^.

Para a análise de dados, todas as variáveis categóricas foram descritas por meio da frequência absoluta e relativa para o total de participantes, e separadamente, para cada tipo de serviço realizado (administrativo e operacional). A variável explicativa foi ser um policial do serviço administrativo ou operacional. As demais variáveis investigadas foram consideradas como variáveis respostas. As análises bivariadas entre as variáveis respostas e a explicativa foram realizadas por meio do teste Qui-Quadrado, ou o teste Exato de Fisher. O teste exato de Fisher foi uma alternativa ao teste Qui-Quadrado nos casos em que as frequências esperadas nas células da tabela de contingência foram inferiores a cinco. Adotou-se o nível de significância de 5%. A análise dos dados foi realizada por meio do *software* estatístico *Stata* ^®^ (versão 16.1).

## RESULTADOS

Entre os policiais militares desse estudo (n=482), 77 (16%) pertenciam ao serviço administrativo e 405 (84%) ao serviço operacional. Foram observadas as seguintes predominâncias do total de policiais: sexo masculino (84,6%); faixa etária entre 23 e 38 anos (52,3%); estado civil com companheiro(a) (70,6%); e graduação/bacharelado como maior nível de escolaridade (52,7%) (Tabela 1). A maioria dos policiais possuía entre 11 e 20 anos de serviço efetivo (55,8%) e relatou que, às vezes, precisava elevar a voz devido ao ruído intenso no trabalho (59,0%). Episódios de agressão física com frequência superior a uma vez por mês foram relatados por 7,4% e ameaças de agressão física por 20,5%, ambos tendo o policial como vítima da situação. As ameaças ou agressões à colegas de trabalho foram relatadas por 19,5% dos policiais (Tabela 2).

**Tabela 1:** Análise descritiva e de associação das questões sociodemográficas e relacionadas à voz segundo o serviço realizado pelos policiais militares (Belo Horizonte, 2022)

**_Variáveis_**	**_Total n (%)_**	**_Serviço administrativo_**		**_Serviço operacional_**		**_Valor-p_**
		**_n_**	**_%_**	**_n_**	**_%_**	
_Sexo_						
_Feminino_	_74 (15,4)_	_17_	_22,1_	_57_	_14,1_	_0,074_ _1_ _*_
_Masculino_	_408 (84,6)_	_60_	_77,9_	_348_	_85,9_	
_Faixa etária (em anos)_						
_23 a 38_	_252 (52,3)_	_31_	_40,3_	_221_	_54,6_	_0,021_ _1_ _**_
_39 a 54_	_230 (47,7)_	_46_	_59,7_	_184_	_45,4_	
_Estado civil_						
_Casado ou outra forma de união_	_334 (70,6)_	_50_	_66,7_	_284_	_71,4_	_0,413_^_1_^
_Sem companheiro (a)_	_139 (29,4)_	_25_	_33,3_	_114_	_28,6_	
_Escolaridade_						
_Fundamental ao Ensino Médio_	_131 (27,2)_	_16_	_20,8_	_115_	_28,4_	_0,387_^_1_^
_Graduação_	_254 (52,7)_	_44_	_57,1_	_210_	_51,8_	
_Pós-graduação_	_97 (20,1)_	_17_	_22,1_	_80_	_19,8_	
_“Sinto que tenho que fazer força para minha voz sair” (IRD_^_BR_^_)_						
_Não_	_396 (82,2)_	_64_	_83,1_	_332_	_82,0_	_0,810_ _1_
_Sim_	_86 (17,8)_	_13_	_16,9_	_73_	_18,0_	
_“Minha voz é rouca” (IRD_^_BR_^_)_						
_Não_	_437 (90,7)_	_70_	_90,9_	_367_	_90,6_	_0,936_^_1_^
_Sim_	_45 (9,3)_	_7_	_9,1_	_38_	_9,4_	
_Probabilidade de disfonia (IRD_^_BR_^_)_						
_Reduzida_	_437 (90,7)_	_70_	_90,9_	_367_	_90,6_	_0,241_^_1_^
_Moderada_	_26 (5,4)_	_2_	_2,6_	_24_	_5,9_	
_Elevada_	_19 (3,9)_	_5_	_6,5_	_14_	_3,5_	
_Ausência do trabalho por problema de voz_						
_Não_	_469 (97,3)_	_58_	_100,0_	_254_	_95,1_	_0,101_ _2__*_
_Sim_	_13 (2,7)_	_0,0_	_0,0_	_13_	_4,9_	

1 Teste Qui-quadrado;

2 Teste exato de Fisher;

* valor-p<0,20;

** valor-p<0,05

Legenda: IRD^BR^ = Instrumento de Rastreio da Disfonia; n = frequência absoluta; % = frequência relativa

**Tabela 2:** Análise descritiva e de associação das questões relacionadas ao trabalho segundo o serviço realizado pelos policiais militares (Belo Horizonte, 2022)

**_Variáveis_**	**_Total n (%)_**	**_Serviço administrativo_**		**_Serviço operacional_**		**_Valor-p_**
		**_n_**	**_%_**	**_n_**	**_%_**	
_Tempo efetivo na polícia militar (em anos)_						
_1 a 10_	_134 (27,8)_	_07_	_9,1_	_127_	_31,4_	_<0,001_ _1__**_
_11 a 20_	_269 (55,8)_	_52_	_67,5_	_217_	_53,6_	
_21 a 30_	_79 (16,4)_	_18_	_23,4_	_61_	_15,0_	
_Elevação da voz devido ao ruído forte no trabalho_						
_Nunca/ raramente_	_65 (13,4)_	_17_	_22,1_	_48_	_11,8_	_<0,001_ _1__**_
_Às vezes_	_284 (59,0)_	_52_	_67,5_	_232_	_57,3_	
_Frequentemente_	_133 (27,6)_	_08_	_10,4_	_125_	_30,9_	
_Agressão física pessoal no trabalho_						
_Nunca/ Menos de 1 vez por mês_	_446 (92,6)_	_74_	_96,1_	_372_	_91,9_	_0,141_ _2_ _*_
_Mais de 1 vez por mês_	_36 (7,4)_	_03_	_3,9_	_33_	_8,1_	
_Ameaça de agressão física no trabalho_						
_Nunca/ Menos de 1 vez por mês_	_383 (80,5)_	_73_	_94,8_	_310_	_76,5_	_<0,001_ _2_ _**_
_Mais de 1 vez por mês_	_99 (20,5)_	_04_	_5,2_	_95_	_23,5_	
_Ameaça ou agressão física ao colega de trabalho_						
_Nunca/ Menos de 1 vez por mês_	_388 (80,5)_	_69_	_89,6_	_319_	_78,8_	_0,028_ _1__**_
_Mais de 1 vez por mês_	_94 (19,5)_	_08_	_10,4_	_86_	_21,2_	
_Apoio social no trabalho_						
_Baixo_	_249 (51,7)_	_24_	_31,2_	_225_	_55,6_	_<0,001_ _1__**_
_Alto_	_233 (48,3)_	_53_	_68,8_	_180_	_44,4_	
_Modelo Demanda-Controle_						
_Baixo desgaste_	_100 (20,8)_	_20_	_26,0_	_80_	_19,8_	_0,499_ _1_
_Trabalho passivo_	_99 (20,5)_	_17_	_22,0_	_82_	_20,2_	
_Trabalho ativo_	_150 (31,1)_	_23_	_30,0_	_127_	_31,4_	
_Alto desgaste_	_133 (27,6)_	_17_	_22,0_	_116_	_28,6_	

1 Teste Qui-quadrado;

2 Teste exato de Fisher;

* valor-p<0,20;

** valor-p<0,05

Legenda: n = frequência absoluta; % = frequência relativa

Não houve associação estatisticamente significativa entre ser um policial do serviço operacional e administrativo e o relato de ter que fazer força para a voz sair, ter a voz rouca, a ausência ao trabalho por problema de voz e a probabilidade de disfonia. Três em cada 100 policiais relataram ter faltado ao trabalho por disfonia nos últimos 12 meses, sendo estes do serviço operacional (Tabela 1).

As seguintes variáveis apresentaram associação estatisticamente significativa com o fato de ser policial do serviço operacional ou administrativo: tempo efetivo na PM; elevação da voz devido ao ruído intenso no trabalho; agressão física pessoal; ameaça de agressão física; ameaça ou agressão física ao colega e apoio social no trabalho (Tabelas 1 e 2).

## DISCUSSÃO

Os resultados sugerem que policiais operacionais e administrativos estão expostos a diferentes condições de trabalho. No entanto, observou-se similaridade na frequência dos relatos de esforço para a voz sair e de rouquidão entre os dois grupos: 18% dos policiais operacionais e 17% dos administrativos relataram esforço para a voz sair, e 9% que a voz é rouca. O esforço vocal foi mais frequentemente relatado que a rouquidão, o que pode indicar demandas vocais intensas mesmo sem a presença clara de alteração perceptiva da qualidade vocal.

O esforço vocal é um conceito multidimensional que pode indicar aumento de *loudness,* tensão muscular, e fatores contextuais como ruído de fundo, distância do interlocutor, carga cognitiva e aspectos emocionais^(17)^. Já os sintomas auditivos de rouquidão, voz grossa e voz fraca relacionam-se principalmente ao uso excessivo e prolongado da voz, com sobrecarga no trato vocal^(18)^.

Em relação à probabilidade de disfonia, observou-se uma maior proporção de policiais administrativos com elevada probabilidade (6,5%) em comparação aos operacionais (3,5%), embora sem significância estatística. Já a probabilidade moderada de disfonia foi maior entre os policiais operacionais (5,9%). A hipótese inicial de maior risco para disfonia entre os operacionais não foi confirmada pelos dados, mas os resultados levantam questões relevantes sobre as características distintas do uso vocal entre os dois serviços. Especula-se que, no serviço administrativo, fatores como o uso contínuo da voz em tarefas como o teleatendimento possam contribuir para o risco de distúrbio vocal. Por outro lado, o serviço operacional envolve uso mais intenso da voz em ambientes ruidosos e sob estresse físico e emocional, o que também pode impactar a saúde vocal.

De forma geral, segundo a validação do IRD^BR^, 9% dos policiais desse estudo apresentaram probabilidade moderada/elevada de disfonia e precisavam realizar uma avaliação vocal completa para a confirmação do diagnóstico de disfonia. Estudo realizado com 442 bombeiros encontrou uma probabilidade moderada/elevada de disfonia de 6,12%^(19)^. O IRD^BR^ utilizado nestes estudos é sensível para captar a probabilidade de disfonia, apresentando um elevado índice de acerto na classificação dessa condição em comparação com outros instrumentos de autoavaliação para rastreamento de disfonia^(13)^. A prevalência de disfonia encontrada nesse estudo foi semelhante a de outros estudos realizados com os adultos norte-americanos^(20,21)^. No entanto, os instrumentos utilizados para o rastreamento de disfonia foram diferentes, e a comparação dos resultados deve ser feita com cautela.

Foi predominante para os policiais no serviço operacional o menor tempo de serviço, a maior exposição à ameaça ou agressão física, a elevação de voz na presença de ruído intenso e o menor apoio social no trabalho.

O resultado mostrou maior proporção de policiais administrativos com tempo efetivo na PM acima de dez anos e de policiais operacionais com menos tempo na carreira. O maior desgaste de saúde do militar, bem como a evidência temível de agressão pessoal no trabalho operacional^(3)^ podem justificar esse achado. Estudo verificou a tendência de os policiais serem realocados para o serviço administrativo dado a desgastes físicos e psicológicos no serviço operacional^(7)^. Nossos resultados refletem um ambiente de trabalho mais estável e menos exigente fisicamente para policiais no serviço administrativo, diferentemente do serviço operacional, que está associado a maior exposição à condições adversas. Tais características do serviço administrativo podem ser mais atraentes para as mulheres e para policiais com mais tempo de carreira por questões de gênero e maior desgaste físico e mental no decorrer dos anos de trabalho^(7)^.

Este estudo indicou que a necessidade de elevar a voz frequentemente devido ao ruído intenso no ambiente de trabalho foi maior para os policiais do serviço operacional. O ruído no ambiente de trabalho pode influenciar negativamente à saúde vocal, especialmente quando combinado com outros fatores de risco individuais e organizacionais^(22)^. Diante da exposição ao ruído elevado das ruas, os militares apresentam uma categoria profissional de maior risco para desenvolver perda auditiva e zumbido^(23)^, assim como os fatores de idade, o tempo de serviço e a exposição a tiros também são preditivos para o adoecimento da saúde auditiva^(24)^.

Nessa perspectiva, falar alto na presença de ruído intenso mostrou-se mais frequente no serviço operacional, tendo em vista a interferência nas ordens verbais e na comunicação em geral do policial militar. O presente estudo não teve como objetivo analisar a relação entre voz e ruído, mas identificou uma prevalência de 30,9% de necessidade frequente de elevar a voz na presença de ruído no trabalho relatada pelos policiais do serviço operacional, em contraste a 10,4% dos policiais no serviço administrativo.

Fatores relacionados ao estresse no trabalho, analisados segundo o modelo demanda-controle, têm sido prioritários nos inquéritos epidemiológicos que investigam a relação saúde-trabalho^(25)^. Esse estudo não encontrou associação estattisticamente signficativa quanto ao modelo demanda-controle segundo o tipo de serviço realizado pelo policial. Porém, a proporção de policiais com alto desgaste (29%) foi maior no serviço operacional quando comparado ao administrativo (22%).

Estudo com policiais operacionais mostrou que a natureza das demandas desse serviço é mais intensa e desgastante, especialmente entre os profissionais de baixa graduação hierárquica^(26)^. Nesse contexto, a presença de apoio social no trabalho, seja por parte dos colegas ou dos chefes e supervisores, exerce influência sobre a saúde e a satisfação profissional, sendo considerada essencial para a manutenção e a promoção da saúde dos trabalhadores^(27)^. Em consonância com essas evidências, nossos resultados indicaram baixos níveis de apoio social, principalmente entre os policiais em serviço operacional, o que pode contribuir para o agravamento das condições psicossociais vivenciadas por esse grupo.

Estudo verificou que policiais no serviço operacional apresentam maior prevalência de estresse ocupacional, faltam mais ao serviço, possuem menores vínculos sociais, entre outros comportamentos de adoecimento do profissional^(28)^. A relação entre as demandas de trabalho de bombeiros e a morbidade psiquiátrica foram atenuadas na presença de altos níveis de apoio social e controle do trabalho^(28)^. Professores com baixo apoio social no trabalho apresentaram mais chance de ter limitação no trabalho por causa da voz do que àqueles com alto apoio social no trabalho^(29)^.

Neste estudo, observou-se baixa frequência de ausência ao trabalho por problema de voz, restrita apenas aos policiais no serviço operacional. A prevalência de faltas foi inferior ao de adultos da população geral (9,2%) no período de referência de 12 meses^(20)^. Outro estudo mostrou que 80% dos teleoperadores de uma central de atendimento a emergências que faltaram ao trabalho por problema de voz em algum momento da carreira, apresentaram queixa vocal^(9)^. É possível que policiais militares continuem trabalhando mesmo apresentando disfonia, o que pode resultar em uma busca tardia por ajuda profissional (fonoaudiólogo ou otorrinolaringologista), agravando o problema ao longo do tempo. O agravamento da disfonia pode dificultar o trabalho dos policiais que estão no serviço operacional, principalmente para as atividades com maior demanda vocal.

A exposição à situação de violência no trabalho foi mais prevalente para os policiais no serviço operacional como era esperado, com maior destaque para a ameaça de agressão física no trabalho (23,5%), o indica um fator de maior estresse desse grupo e que pode gerar a necessidade de elevar a intensidade de voz e aumentar a tensão corporal. Estudo com professores da educação básica evidenciou relação entre a limitação no trabalho por causa da voz autorreferida e o fato de terem sido expostos à violência verbal praticada pelos estudantes^(29)^. A autorreferência sobre distúrbios de voz não apresentou associação estatisticamente significativa com as situações de violência em outro estudo com professores^(30)^.

Ressalta-se que o presente estudo foi realizado com uma amostra não- probabilística, ou seja, os resultados não podem ser generalizados para a população-alvo. Os resultados indicam a necessidade de maior compreensão sobre a influência do ruído no trabalho, ameaça ou agressão física e apoio social no trabalho, como possíveis fatores de risco para a disfonia entre policiais militares. Essa questão deve ser explorada em estudos futuros para elucidar a relação desses fatores com a saúde vocal dessa categoria profissional. Além disso, a menor proporção, na amostra desse estudo, de policiais no serviço administrativo reflete a composição da própria Polícia Militar de Minas Gerais, Brasil, e não uma falha amostral. O tamanho das amostras desiguais entre os grupos (administrativos e operacionais) pode implicar limitações interpretativas. Entretanto, acredita-se que a análise oferece contribuições relevantes para compreender as diferenças nas condições de trabalho e na probabilidade de disfonia entre os policiais nos dois tipos de serviço.

O efeito do trabalhador sadio é um viés comum em estudos ocupacionais^(31)^ e pode ser relevante na interpretação dos resultados dessa pesquisa. Ele pode ocorrer porque policiais menos saudáveis ou com condições que os impedem de exercer atividades mais exigentes fisicamente tendem a ser transferidos para o serviço administrativo ou se afastam do trabalho completamente, enquanto os mais saudáveis permanecem ou são alocados para o serviço operacional. Esse fator pode ter influenciado os resultados relacionados à voz observados neste estudo, considerando que os sintomas vocais podem refletir diversas condições de saúde, como alterações respiratórias, gástricas, mentais, entre outras^(32)^.

Os resultados desse estudo não possuem validade externa e devem ser interpretados com cautela. Policiais militares não são tradicionalmente reconhecidos como profissionais da voz falada, o que pode levar à subvalorização ou à não identificação de sintomas vocais por parte desses trabalhadores. Além disso, observa-se uma tendência de migração dos policiais em serviço operacional para funções administrativas ao longo da carreira. Esses fatores podem impactar a detecção de diferenças na probabilidade de disfonia entre policiais em serviços operacional e administrativo.

Este estudo apresentou como limitação o fato de o questionário ter sido enviado aos participantes pela instituição militar, o que dificulta o controle do método de aplicação. Estudos por meio de *Websurvey* apresentam vantagens como a possibilidade de coletar dados a distância, menor custo e maior facilidade de implantação^(33)^. Embora tenha sido possível assegurar o número exato de usuários que foram alcançados, não foi obtida a informação sobre quantos visualizaram o e-mail enviado. A taxa de resposta foi reduzida e não foi possível realizar a avaliação de não-resposta, ou seja, as informações necessárias para responder se os usuários que não participaram são semelhantes aos que participaram^(33)^. Por outro lado, a utilização de questionários validados previamente foi uma excelente decisão na busca de consistência e medidas acuradas. Outro ponto positivo, foi a testagem da versão on-line do questionário antes do início do estudo, tanto para avaliar a compreensibilidade quanto a facilidade e tempo de resposta^(33)^.

Os resultados indicaram a presença de estressores ocupacionais que podem ou não ter associação com a disfonia do policial militar. Futuros estudos devem buscar analisar os fatores associados à disfonia em policiais militares administrativos e operacionais, considerando as condições de trabalho distintas entre os grupos. Ações de prevenção de disfonia e de promoção da saúde vocal para os policiais militares são necessárias, com foco na conscientização sobre os fatores de risco para a disfonia e no aconselhamento de como evitá-los ou minimizá-los. Tais ações devem estar acompanhadas de medidas institucionais que melhorem as condições de trabalho, como o controle do ruído ambiental, a valorização do apoio social nas equipes, a adequação da carga de trabalho e a oferta de treinamentos sobre o uso saudável da voz no contexto das atividades policiais.

## CONCLUSÃO

Os policiais militares no serviço operacional possuem menor tempo de serviço, maior relato de exposição à ameaça ou agressão física, da elevação da voz devido ao ruído intenso no ambiente de trabalho e do baixo apoio social no trabalho, indicando condições de trabalho desfavoráveis para a saúde. Esses fatores podem contribuir para o desenvolvimento ou agravamento de alterações vocais, bem como para outros agravos relacionados à saúde.

Houve similaridade na frequência dos relatos de esforço para a voz sair e de rouquidão, além de reduzido número de faltas ao trabalho por problema vocal em policiais de ambos os serviços. A probabilidade de disfonia moderada/elevada foi similar para o policial em serviço operacional e administrativo e indicou que 9% dos policiais devem realizar a avaliação vocal completa para confirmação do diagnóstico.
